# Proliferation extent of CD34^+^ cells as a key parameter to maximize megakaryocytic differentiation of umbilical cord blood-derived hematopoietic stem/progenitor cells in a two-stage culture protocol^☆^

**DOI:** 10.1016/j.btre.2014.07.002

**Published:** 2014-07-16

**Authors:** Javad Hatami, Pedro Z. Andrade, Denise Bacalhau, Fernando Cirurgião, Frederico Castelo Ferreira, Joaquim M.S. Cabral, Cláudia L. da Silva

**Affiliations:** a Department of Bioengineering and IBB – Institute for Biotechnology and Bioengineering, Instituto Superior Técnico, Universidade de Lisboa, Av. Rovisco Pais, Nr. 1, 1049-001 Lisbon, Portugal; b Department of Obstetrics, S. Francisco Xavier Hospital, Estrada do Forte do Alto do Duque, 1449-005 Lisbon, Portugal

**Keywords:** Megakaryocytic differentiation, Platelets, Megakaryocytes, Hematopoietic stem/progenitor cells, Umbilical cord blood

## Abstract

Co-infusion of *ex-vivo* generated megakaryocytic progenitors with hematopoietic stem/progenitor cells (HSC/HPC) may contribute to a faster platelet recovery upon umbilical cord blood (UCB) transplantation. A two stage protocol containing cell expansion and megakaryocyte (Mk) differentiation was established using human UCB CD34^+^-enriched cells. The expansion stage used a pre-established protocol supported by a human bone marrow mesenchymal stem cells (MSC) feeder layer and the differentiation stage used TPO (100 ng/mL) and IL-3 (10 ng/mL). 18% of culture-derived Mks had higher DNA content (>4 N) and were able to produce platelet-like particles. The proliferation extent of CD34^+^ cells obtained in the expansion stage (FI-CD34^+^), rather than expansion duration, determined as a key parameter for efficient megakaryocytic differentiation. A maximum efficiency yield (EY) of 48 ± 7.7 Mks/input CD34^+^ cells was obtained for a FI-CD34^+^ of 17 ± 2.5, where a higher FI-CD34^+^ of 42 ± 13 resulted in a less efficient megakaryocytic differentiation (EY of 22 ± 6.7 and 19 ± 4.6 %CD41).

## Highlights

• A two-stage protocol established aiming at effective Mk differentiation of UCB CD34^+^-enriched cells.
• Proliferation extent of CD34^+^ cells during expansion identified as a key parameter to maximize Mk differentiation.
• Morphological analysis demonstrated the characteristic features of *ex*-*vivo* generated Mks and platelet-like particles.

## 1 Introduction

Allogeneic transplantation of CD34^+^-enriched cells from human umbilical cord blood (UCB) as a source of hematopoietic stem/progenitor cells (HSC/HPC) is a potential therapy for treating hemato-oncological diseases and other blood disorders in adult patients [1], [2]. However, a delayed platelet recovery is typically associated to the transplantation of HSC/HPC from UCB, when compared to adult sources (bone marrow (BM) and mobilized peripheral blood (mPB)) [3]. Administration of *ex-vivo* generated megakaryocytic progenitor cells and megakaryocytes (Mks) alone or co-infusion with UCB HSC/HPC can be a promising strategy to reduce the prolonged period of platelet recovery [4], [5].

Mks are rare, large and polyploid myeloid cells, which reside primary in the BM region adjacent to sinusoidal walls [6]. Platelet biogenesis from Mks occurs through nuclear polyploidization, cellular enlargement, cytoplasmic maturation and platelet release. The production of Mks and platelets from different sources of cells such as UCB, BM or mPB, as well as embryonic stem cells and induced pluripotent stem cells has been studied over the last decades [7]. In this context, different biological, chemical and physical factors have been studied in order to establish an optimal protocol to enhance megakaryocytic differentiation from primitive cell populations [8], [9], [10], [11].

The main objective of this study was to test if an optimized expansion stage followed by a megakaryocytic differentiation stage would be an effective strategy to maximize Mk production from UCB HSC/HPC. Specifically, we aimed at systematically identifying a relation between proliferation extent of CD34^+^ cells and effective megakaryocytic differentiation.

## 2 Material and methods

### 2.1 Cell culture

hUCB and hMSC samples were obtained from healthy donors after maternal donor and donor informed consent, respectively. CD34^+^-enriched cells from UCB were expanded using a previously optimized protocol [12]. Briefly, low density mononuclear cells (MNC) were separated from UCB (more than 9 UCB units from individual donors) by Ficoll density gradient (1.077 g/mL; GE Healthcare) and then enriched for CD34^+^ antigen by magnetic activated cell sorting (MACS; Miltenyi Biotec). UCB CD34^+^-enriched cells (ranging 70–90% CD34^+^ cells) were co-cultured (3.0 × 10^3^ cells/mL, 5 mL) with BM mesenchymal stem cell (BM-MSC) feeder layer. BM-MSC was previously cultured (totally from 3 different individual donors, passage 3–6) using Dulbecco's modified essential medium (DMEM; Gibco) plus 10% fetal bovine serum (FBS; Gibco) until confluence and then inactivated with mitomycin C (0.5 μg/mL, Sigma) to prevent cell overgrowth. Serum-free QBSF-60 culture medium (Quality Biological Inc.) supplemented with SCF (60 ng/mL), Flt-3L (55 ng/mL), TPO (50 ng/mL) and b-FGF (5 ng/mL) (all from Peprotech) was used in the expansion stage [12]. Expanded cells were differentiated toward Mk lineage at density of 2.0 × 10^5^ cells/mL (totally in 1 mL) in Iscove's modified Dulbecco's medium (IMDM) supplemented with 10% FBS, 1% penicillin–streptomycin and 0.1% Fungizone (all from Gibco). The effect of different concentrations and combinations of IL-3 (10 ng/mL) and TPO (30, 50 and 100 ng/mL; both from Peprotech) were evaluated. At days 3, 5 and 7 of differentiation stage, half of exhausted medium was replaced by the fresh medium containing the same concentration of cytokines. Cell numbers were determined by the trypan blue (Gibco) dye exclusion method and they were reported by considering the number of expanded cells cultivated in the differentiation stage.

### 2.2 Cell characterization

(i) In order to assess the degree of megakaryocytic differentiation, CD41 (Mk lineage cells) expression was analyzed by flow cytometry (FACSCalibur, BD) using an anti-CD41 antibody (Biolegend). CD34 and CD33 expression was also determined using appropriate antibodies and isotype controls. (ii) Mk ploidy was determined by double-staining technique with flow cytometry (FACSCalibur, BD) and using CellQuest Pro software (BD) by choosing CD41^+^ events as a respected gate [13]. Briefly, the cell cultures incubated 15 min with anti-CD41 antigen (Biolegend) and then fixed by 70% cold ethanol (4 °C). Cells were re-suspended in a staining solution containing propidium iodide (50 μg/mL; Sigma), sodium citrate (4 mM; Sigma), RNase A (0.1 mg/mL; Sigma), Triton X-100 (0.1%; Sigma) in pH 7.8 1 h before performing the flow cytometry.

### 2.3 Electron microscopy

(i) For scanning electron microscopy imaging, cell population were first fixed in a solution of glutaraldehyde (Sigma) 1.5% (v/v) in PBS (Gibco), then post-fixed in a solution of osmium tetroxide (0.05%; Sigma) in PBS (Gibco); both for 30 min at room temperature. Cells were then dehydrated by gradually increase of ethanol (Sigma) concentration (50%, 75% and 100% in distilled water). Finally, cell populations were coated with gold and observed using scan electron microscope (Hitachi S2400). (ii) In order to observe internal structure of Mks by transmission electron microscopy (TEM), culture-derived cells were fixed in a solution containing 2% paraformaldehyde (Sigma) and 2.5% glutaraldehyde (Sigma) in 0.1 M sodium cacodylate buffer (Sigma) (pH 7.4) for 1 h at room temperature (22 °C). After rinsing with cacodylate buffer (Sigma), cells were post-fixed with a 1% osmium tetroxide (Sigma) in 0.1 M cacodylate buffer (Sigma) for 1 h at room temperature. Cells were then fixed with uranyl acetate (Sigma) (0.5%) in citrate–acetate acid buffer (pH: 5–6) and dehydrated by graduate increasing ethanol (Sigma) concentration (50%, 75% and 100% in distilled water). Finally, cell populations were embedded in Epon (Sigma), cut and Mks ultrastructure observed with TEM apparatus (Hitachi 8100).

### 2.4 Statistical analysis

Results are presented as a mean ± standard error of mean (SEM). Results were statistically analyzed using two-sided non-paired Student's *t*-test by Microsoft Excel and considered significant when *p* < 0.05.

## 3 Results and discussion

### 3.1 Impact of the proliferation extent of CD34^+^ cells on megakaryocytic differentiation of UCB CD34^+^-enriched cells

**Fig. 1:**
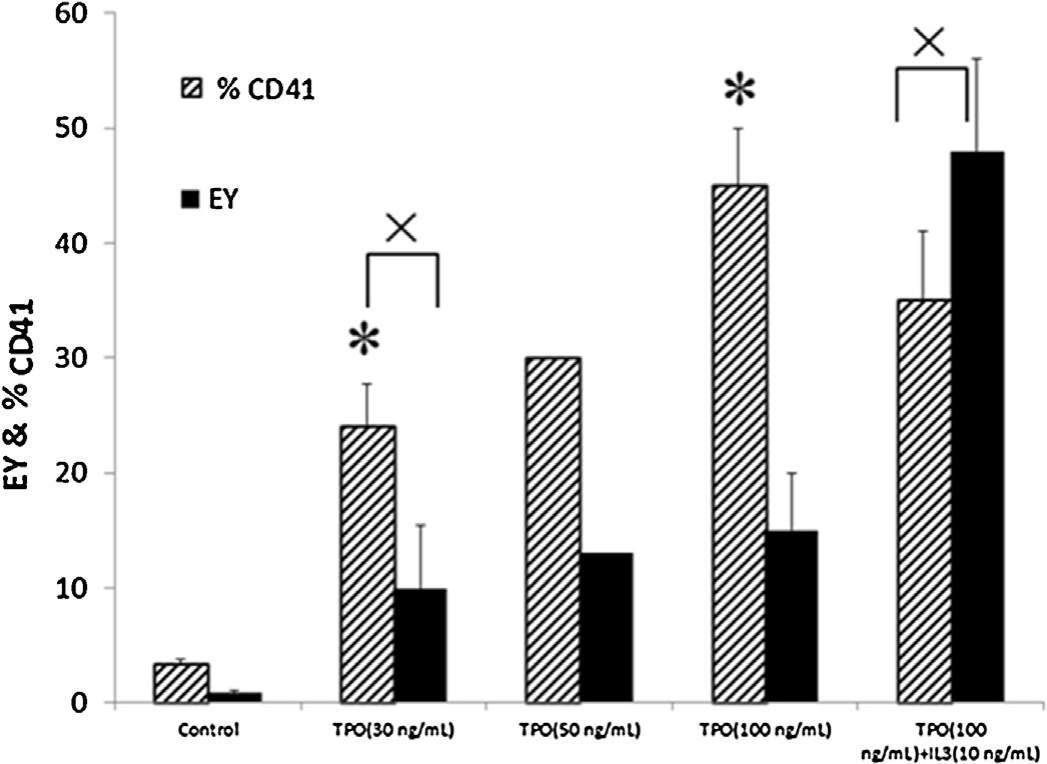
Effect of different TPO concentrations and combination with IL-3, used in the differentiation stage, on EY and %CD41. (* – *p* < 0.04 and × – *p* < 0.05).

CD34^+^-enriched cells from UCB were expanded using a previously optimized protocol [12] and differentiated toward Mk lineage using a simple protocol containing only two cytokines (TPO and IL-3). Expanded cells were also differentiated, as a control, using the same protocol but without any supplemented cytokines. The effect of addition of TPO and IL-3 on Mk differentiation is illustrated in Fig. 1.

Efficiency yield of the whole process (EY) was determined according to Eq. (1). Therefore, EY is a measure on how many megakaryocytic cells can be produced from each initial single UCB CD34^+^-enriched cell seeded to the expansion stage.

(1) $$\mathrm{EY}=\frac{\text{number}\text{of}{\mathrm{CD}41}^{+}\text{cells}(\text{at }\text{the}\text{end}\text{of}\text{differentiation}\text{stage})}{\text{number}\text{of}{\mathrm{CD}34}^{+}\text{cells }(\text{seeded }\text{to }\text{the}\text{expansion}\text{stage})}$$

The result presented in Fig. 1 demonstrated that the higher percentage of CD41^+^ cells can obtained by increasing concentration of TPO (*p* < 0.04 for 100 ng/mL compared to 30 ng/mL). However, increasing TPO concentration alone, from 30 to 100 ng/mL, was not enough to stimulate a simultaneous cell differentiation and proliferation at high levels. The combination of TPO (100 ng/mL) and IL-3 (10 ng/mL) lead to a significant increase in EY and %CD41, when compared to the control (*p* < 0.05 for both parameter). The introduction of IL-3 at a low concentration (10 ng/mL), together with TPO (100 ng/mL), allowed to increase 3.2 times the total EY of the process (*p* < 0.05), though such increase in EY was obtained on the expense of CD41 purity, corresponding to a slight, but statistically significant 10% decrease (*p* < 0.05) in %CD41. Considering these results, the following experiments were performed using TPO (100 ng/mL) and IL-3 (10 ng/mL) in the differentiation stage.

**Fig. 2:**
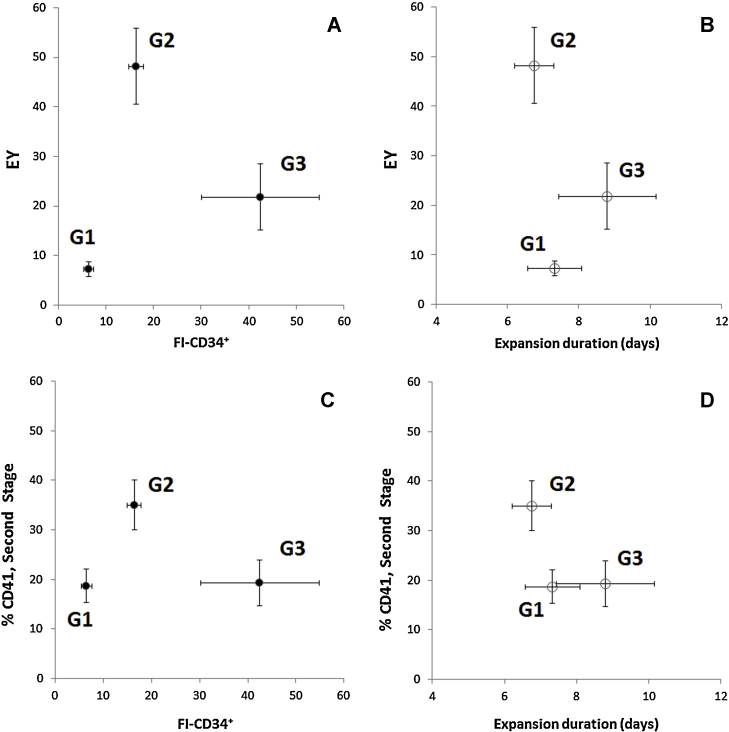
Effect of UCB CD34^+^-enriched cell's expansion stage, co-cultured with a feeder layer, on megakaryocytic differentiation: EY (A) and %CD41 (C) were evaluated with respect to FI-CD34^+^ (EY and FI-CD34^+^ were significantly different among all groups, *p* < 0.05). EY (B) and %CD41 (D) were evaluated with respect to the expansion duration (no significant differences between groups, *p* > 0.3). %CD41 was significantly different between G2 and either G1 or G3, *p* < 0.05), *n* ≥ 4 for each group.

In the present study we were able to quantitatively determine the relation between the extent of proliferation of CD34^+^ cells, assessed as fold increase in CD34^+^ cells (FI-CD34^+^) and final Mk production (EY and %CD41 in Fig. 2A and C, respectively). FI-CD34^+^ was calculated according to Eq. (2).

(2) $${\text{FI-}\mathrm{CD}34}^{+}=\frac{\text{number}\text{of}{\mathrm{CD}34}^{+}\text{cells}(\text{at}\text{the}\text{end}\text{of}\text{expansion}\text{stage})}{\text{number}\text{of}{\mathrm{CD}34}^{+}\mathrm{cells}(\text{seeded}\text{to}\text{the}\text{expansion}\text{stage})}$$

Cell populations were grouped in order to individualized distinct relations between EY and FI-CD34^+^. The criteria used for such population grouping were to minimize SEMs associated to both EY and FI-CD34^+^, but more importantly to obtain statistically significant differences between such groups (*p* < 0.05). Considering these criteria, the best possible grouping (G1, G2 and G3) was represented in Fig. 2A and C. FI-CD34^+^ obtained for G1, G2 and G3 were 6.5 ± 1.0, 17 ± 2.0 and 42 ± 7.1, respectively, corresponding to 0.85 ± 0.11 × 10^6^, 2.1 ± 0.16 × 10^6^ and 4.8 ± 0.77 × 10^6^ CD34^+^ cells at the end of the expansion stage. The G1 group with the lowest FI-CD34^+^ has the lowest EY of 7.3 ± 1.5 (0.98 ± 0.21 × 10^6^ CD41^+^ cells at end of differentiation stage).

Comparison between G3 and G2 results points out that despite the higher FI-CD34^+^ obtained for G3, a lower EY (22 ± 4.7 vs. 49 ± 3.7; *p* < 0.05), a lower %CD41 (19 ± 4.6% vs. 36 ± 3.8%; *p* < 0.05) and a lower number of CD41^+^ cells (2.7 ± 0.63 × 10^6^ vs. 6.0 ± 0.67 × 10^6^; *p* < 0.05) were obtained, by the end of the differentiation, for G3 when compared to G2. Therefore, these results demonstrated that an over-expansion of CD34^+^ cells led to a loss of Mk commitment potential of the expanded cells. This observation is highlighted when two populations of CD34^+^-enriched cells from the same UCB were cultured; one of this populations was expanded with a FI-CD34^+^ in the range of G2 (FI-CD34^+^:15.1) and another one in the range of G3 (FI-CD34^+^:60). The first experiment resulted in EY of 67 with 25% CD41^+^ cells though the second experiment resulted in a lower EY and %CD41 (38 and 5.9%, respectively). Considering that the FI-CD34^+^ is related with cell population doublings, in an idealized cell population, a FI-CD34^+^ of 16 and 64 would correspond to 4 and 6 cell population doublings, respectively. Different factors can contribute to the loss of Mk differentiation potential for G3, namely cell commitment toward the granulocytic and monocytic lineage (24.0 ± 4.3% CD14^+^ cells) [12], or neutrophil lineages (64.0 ± 12.1% HLA-DR^++^ CD117^++^) [14].

As a control, UCB CD34^+^-enriched cells were expanded in the same culture conditions, but in absence of feeder layer; regardless the different conditions tested, both FI-CD34^+^ and EY were maintained at low levels (2.7 ± 0.91 and 7.0 ± 1.2, respectively; *n* = 3). It has been previously reported that FI-CD34^+^ was consistently lower in the absence of feeder [12], [15]. Therefore, this result highlighted the positive effect of presence of feeder layer, in the expansion stage, when targeting an efficient Mk differentiation.

Boyer and colleagues have previously suggested a 5-day expansion period as optimal for the increased production of Mks from UCB CD34^+^ cells (>95% enriched) in a two-phase protocol. However, using FI-CD34^+^ in the expansion stage as an operational parameter, rather than the expansion duration, has more advantages such as considering the intrinsic biological variability of UCB samples and the impact of initial CD34^+^ enrichment. The current study thus demonstrated that by using FI-CD34^+^, as a key parameter, we were able to determine the effectiveness of megakaryocyte differentiation of UCB cells, identifying different groups with statistical significance (G1, G2 and G3 in Fig. 2A and C in terms of FI-CD34^+^, *p* < 0.05). Indeed, such identification would not be statistically significant if expansion duration was used instead (Fig. 2B and D; *p* > 0.3 between G1, G2 and G3 in terms of expansion duration).

**Fig. 3:**
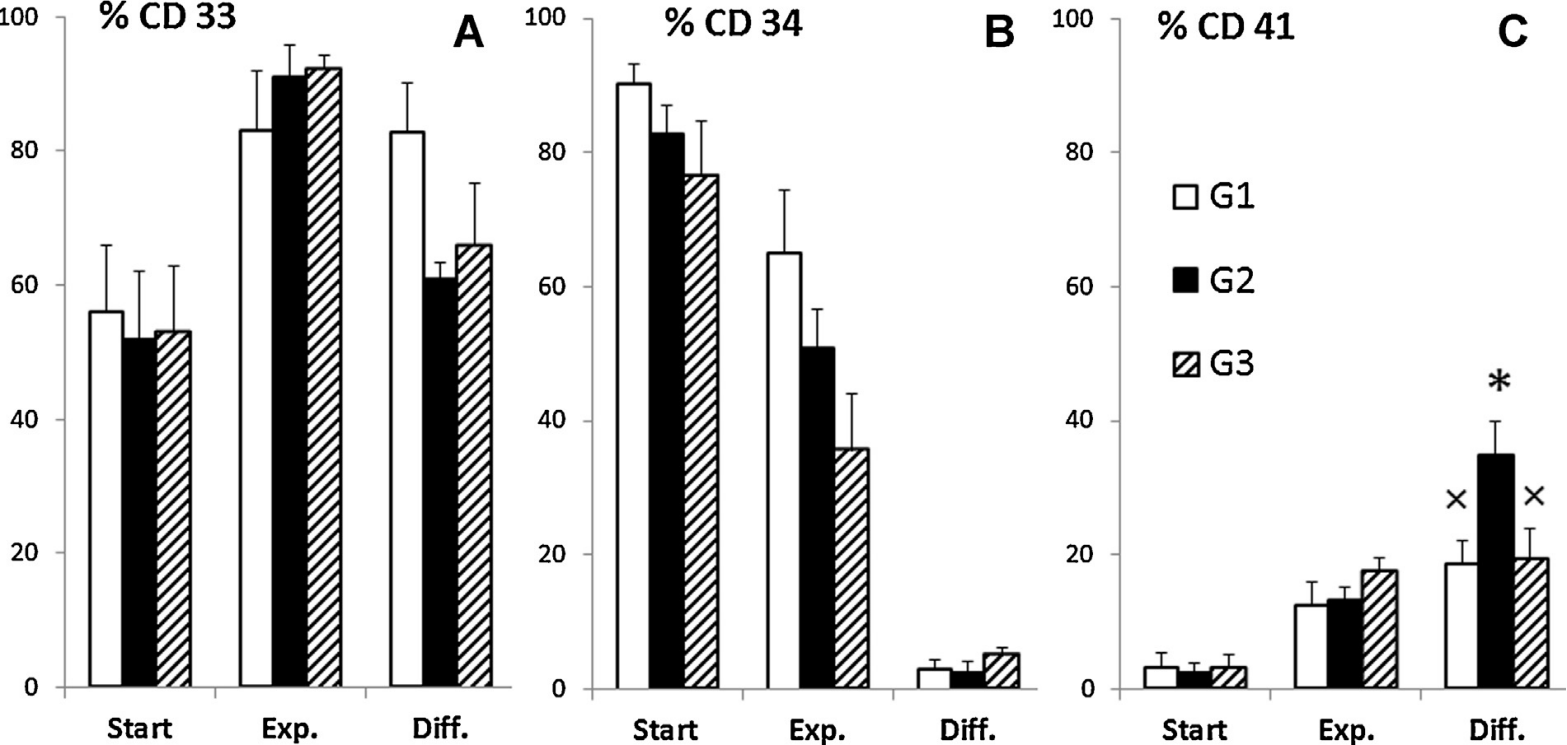
Phenotypic characterization of the UCB CD34^+^-enriched population; %CD33 (A), %CD 34 (B) and %CD41 (C) of each group at the start of expansion (start), the end of expansion (Exp.) and differentiation (Diff.) stages. Results presented as mean ± SEM (*n* ≥ 4) and *p* < 0.05 between * and ×.

In the current study, the initial population consisted of 1.5 × 10^5^ cells with similar cell population compositions (Fig. 3). At the end of the expansion, the total numbers of cells were 1.7 ± 0.40 × 10^6^, 4.2 ± 0.30 × 10^6^ and 20 ± 9.1 × 10^6^ for G1, G2 and G3, respectively. In the expansion stage, the reduction in %CD34 (from 90 to 65% for G1, from 83% to 51% for G2 and from 77% to 36% for G3) was accompanied by an increase in %CD33 (early myeloid cells), from 56% to 83% for G1, from 52% to 91% for G2 and from 53% to 92% for G3.

A significant decrease in %CD34 was observed during the differentiation stage (from 65% to 2.9% for G1, 51–2.5% for G2 and 36–5% for G3, Fig. 3A and B). Such decrease in %CD34 was concomitant to an increase in %CD41 indicating differentiation toward Mk lineage. Comparing with the expansion stage, %CD41 increased during differentiation stage from 13% to 19% for G1, while for G2 raised from 13% to 35%, but only from 17% to 19% for G3 (Fig. 3C). Over differentiation stage, the total number of cells increased about 3.7 folds for G1 (corresponding to 6.3 ± 1.0 × 10^6^ total cells), and 4.4 folds for G2 (corresponding to 19 ± 4.2 × 10^6^ total cells), but only about 1.3 for G3 (corresponding to 26 ± 13 × 10^6^ total cells).

### 3.2 Characterization of cultured-derived Mks and platelets-like particles

**Fig. 4:**
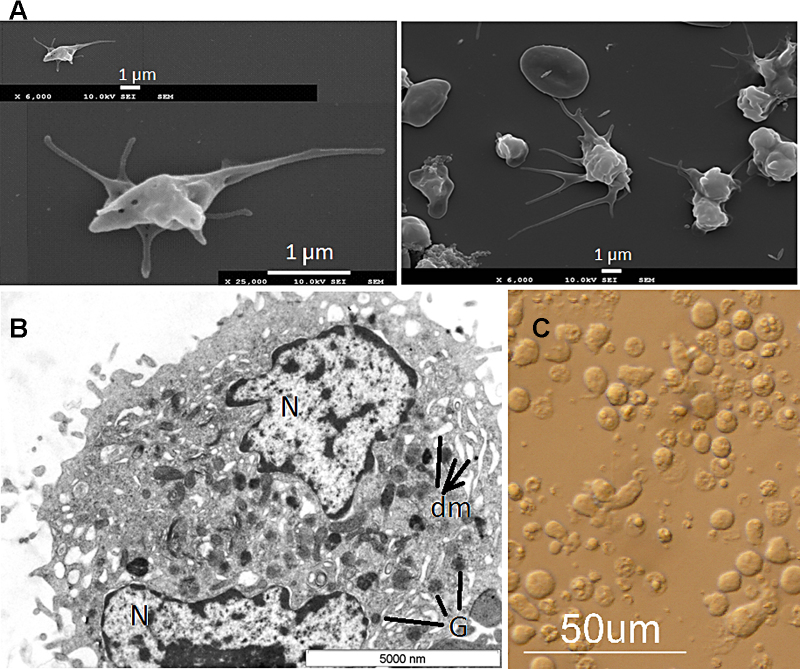
Microscopy imaging: representative scanning electron microscopy images of human peripheral blood-derived platelets (left) and *ex-vivo* generated UCB-derived platelet-like particles (right) (A), transmission electron microscopy (TEM) images of culture-derived Mk. The nucleus (N), demarcation membrane (dm) and α-granules (G) are indicated in the image (B). Morphology of culture-derived cells by inverted microscopy (C). All images were drawn from G2 group.

Scanning electron microscopy analysis showed similar morphology of culture-derived platelet-like particles and human PB-derived platelets (Fig. 4A, right and left, respectively), demonstrating the ability of the current protocol to support the in vitro production of platelet-like particles. Likewise, transmission electron microscopy (TEM) analysis of culture-derived Mk (Fig. 4B) showed normal features of a mature Mks with demarcation membrane (dm) system, nucleus (N) and α-granules characteristic of such mature Mk. Electron microscopy (SEM and TEM) imaging was performed on 3 different populations from G2 and for each culture, platelet-like particles (similar to the Fig. 4A) was identified in more than 10 microscopy images.

**Fig. 5:**
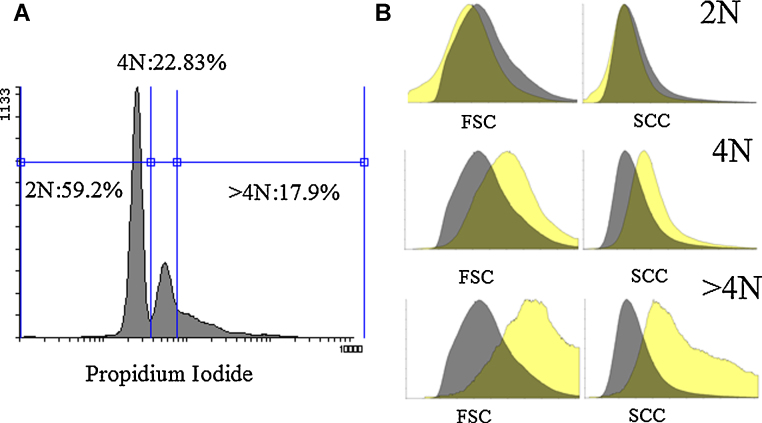
Ploidy analysis of culture-derived Mks (A), forward (FSC) and side (SCC) scatter properties of CD41^+^ cells with 2 N, 4 N and >4 N DNA content (light-yellow) (B). Background scatters (dark-grey) belong to all CD41^+^ cell population. (For interpretation of the references to color in this figure legend, the reader is referred to the web version of this article.)

Ploidy analysis (Fig. 5A) revealed that about 18% of culture-derived Mks have higher ploidy (>4 N). Moreover, forward (FCS) and side scatter (SCC) properties of such population are higher compared to the CD41^+^ cells with 2 N and 4 N DNA content (Fig. 5B). Mks generated from UCB, compared to human PB, were described to be smaller and have less ploidy; however, as reported previously [13] and confirmed in the current study, these are still able to produce platelets-like particles.

## 4 Conclusions

The current study presents a two-stage protocol aiming at effective megakaryocytic differentiation of UCB CD34^+^-enriched cells. The results identified distinct individual groups which elucidate the relation between FI-CD34^+^ and efficiency of Mk production. This information is valuable to balance the proliferation and differentiation potential of CD34^+^ cells, when targeting efficient Mk production. The underlying phenomena for such balance should be actually based in cell population doublings, but FI-CD34^+^ is a tangible parameter easier to quantify. Several studies have reported production of Mk cells and platelets from HSC/HCP. For example, using UCB progenitors, a perfusion system was used to produce enough number of platelets in vitro for clinical transfusion (300–600 × 10^9^) [16]. However, the drawback of aforementioned work was most of culture-derived platelets were activated in the absence of any agonists. Another study reported producing 44 ± 8.1 Mks/input HSC/HPC using human mPB cells through a complex 3-step culture; includes a cocktail comprised by 17 different cytokines and changes in pH and O_2_ tension during experiment [17]. However, by using only two cytokines (TPO and IL-3), in the differentiation stage, our simpler differentiation protocol was able to produce 48 ± 7.7 Mks/input CD34^+^ cells using a 17 ± 2.5 FI-CD34^+^ and benefits from using UCB progenitors which are largely available and usually discarded after delivery involving a non-invasive collection procedure. This work quantitatively demonstrates that the FI-CD34^+^, rather than expansion duration, can be used as a key parameter to maximize Mk cell generation from CD34^+^-enriched cells. When adapted to fully defined, GMP-compliant culture reagents and conditions, this protocol has the potential to impact cellular therapies within the hemato-oncological field.

## Conflict of interest

The authors declare no commercial or financial conflict of interest.
